# Concomitant aortic regurgitation predicts better left ventricular reverse remodeling after transcatheter aortic valve replacement

**DOI:** 10.1186/s12872-023-03377-7

**Published:** 2023-07-17

**Authors:** Hao-Ran Yang, Tian-Yuan Xiong, Yi Zhang, Jing-Jing He, Yuan Feng, Mao Chen

**Affiliations:** Department of Cardiology and Laboratory of Heart Valve Disease, West China Hospital, Sichuan University, #37 Guoxue Alley, Chengdu, 610041 PR China

**Keywords:** Transcatheter aortic valve replacement, Left ventricular reverse remodeling, Aortic regurgitation, Epicardial adipose tissue

## Abstract

**Background:**

We aim to determine predictors of inadequate left ventricular mass index (LVMi) regression at mid-term after transcatheter aortic valve replacement (TAVR), including the potential role of epicardial adipose tissue (EAT).

**Methods:**

We retrospectively reviewed patients with both echocardiographic assessments and multi-slice computed tomography (MSCT) obtained one year after TAVR. The change of LVMi, the volume and the average CT attenuation of EAT from baseline to one-year follow-up was calculated. Patients were divided into two groups by the percentage change of LVMi at a cut-off of 15%.

**Results:**

A total of 152 patients were included with a median age of 74 years (interquartile range [IQR] 69–78 years) and 56.6% being male. LVMi decreased (P < 0.0001) while EAT volume increased and the average CT attenuation decreased (both P < 0.0001) at one year. Baseline atrial fibrillation (P = 0.042), mitral regurgitation ≥ mild (P = 0.006), aortic regurgitation (AR) > mild (P = 0.001) and the change in the average CT attenuation of EAT (P = 0.026) were different between the decrease of LVMi ≥ 15% and < 15%. AR > mild at baseline was the only statistically significant predictor of a decrease of LVMi < 15% at one year (OR 0.33, 95% CI: 0.13 to 0.84, P = 0.021) in multivariate regression.

**Conclusions:**

Concomitant more-than-mild AR might predict better left ventricular reverse remodeling regression after TAVR.

## Introduction

Left ventricular (LV) remodeling, which has been associated with worse clinical outcomes [1–4],is a common response to pressure overload caused by severe aortic stenosis (AS). The relief of this overload from transcatheter aortic valve replacement (TAVR) tends to provoke LV reverse remodeling, which is demonstrated as a decrease in left ventricular mass index (LVMi). In a previous study, it has been observed that LVMi underwent a rapid decrease in the first year after TAVR with a median percentage of decrease at 14.5% and reached a relative plateau afterwards. [5]. The degree of LVMi regression varies across patients and it could serve as an independent predictor of postoperative outcomes after TAVR in patients without significant paravalvular leakage (PVL) [6]. Given its impact on the prognosis, it is of great importance to ascertain the predictors of LVMi regression after TAVR. Previous studies have identified that male and TAVR-induced left bundle branch block (LBBB) predict less favorable LV reverse remodeling [7, 8]. Besides, it is noteworthy that some studies have found the volume of epicardial adipose tissue (EAT) is associated with LV remodeling in the progression of calcific AS and the clinical outcomes after TAVR [9–11]. However, the change in the volume and the average CT attenuation of EAT after TAVR and their relationship with LV reverse remodeling remain unclear. Herein, we retrospectively reviewed TAVR patients in our center and compared two groups of patients by the degree of LVMi regression at one year, with the aim of determining predictors of inadequate LVMi regression.

## Methods

### Patient population

We retrospectively included patients with severe symptomatic AS who underwent TAVR in our center with both echocardiographic assessments and multi-slice computed tomography (MSCT) obtained one year after TAVR. Patients with failed aortic bioprostheses were excluded. The indication for TAVR was discussed in all cases by our multidisciplinary Heart Team. Baseline and clinical characteristics, echocardiographic and computed tomographic measurements, as well as procedural and post-procedural details were collected in a dedicated prospective TAVR database. This study was approved by the institutional review board and all patients gave written informed consent.

### Procedures

All of the procedures were performed under general anesthesia in the hybrid operating room before September 2016 and in the catheterization laboratory with local anesthesia and conscious sedation afterwards. The transfemoral approach was the default choice of access. Balloon pre- and post-dilation were performed according to operator discretion. Transcatheter heart valves used during the study period were mainly domestic first generation self-expanding devices with no outer skirt or recapturable capability (i.e. Venus-A valve, Venus MedTech, Inc., Hangzhou, China; VitaFlow valve, MicroPort, Shanghai, China). The size of transcatheter heart valve was determined according to the comprehensive analysis of annular dimension and landing zone calcification. A second valve might need to be implanted in the index procedure due to valve migration or significant PVL.

### Echocardiography

Transthoracic echocardiography (TTE) was arranged at baseline and at follow-ups after TAVR. The severity of aortic regurgitation (AR) and mitral regurgitation (MR) was determined by two experienced echocardiographers. The main grading criteria for AR were vena contracta width (CVW) and/or the ratio of the width of AR jet to the diameter of the left ventricular out flow tract, where the CVW < 0.3 cm and/or the ratio < 25% indicated mild AR, 0.3 cm ≤ CVW ≤ 0.6 cm and/or 25%≤ the ratio < 65% indicated the moderate AR, CVW > 0.6 cm and/or the ratio ≥ 65% indicated severe AR [12]. The main criterion for MR was the CVW, where CVW < 0.3 cm indicated mild MR while CVW ≥ 0.7 cm indicated severe MR. [12] When grading severity was difficult by the parameters mentioned above, other qualitative or quantitative parameters were used according to the guidelines [12]. Left ventricular inner diameter in diastole (LVIDd), intraventricular septum in diastole (IVSd), and LV posterior wall in diastole (LVPWd) were measured in parasternal long axis view in accordance with guidelines recommendations [13]. LVM was calculated by the formula LVM = 0.8 × 1.04×([LVIDd + IVSd + LVPWd]^3^− LVIDd^3^) + 0.6 g and divided by body surface area to acquire LVMi. Patients were divided into two groups by the percentage change of LVMi (i.e. [baseline LVMi– 1-year LVMi] / baseline LVMi) at a cut-off of 15% according to the observation that the median percentage decrease of LVMi was 14.5% at one year [5].

### MSCT protocol and measurements

**Fig. 1:**
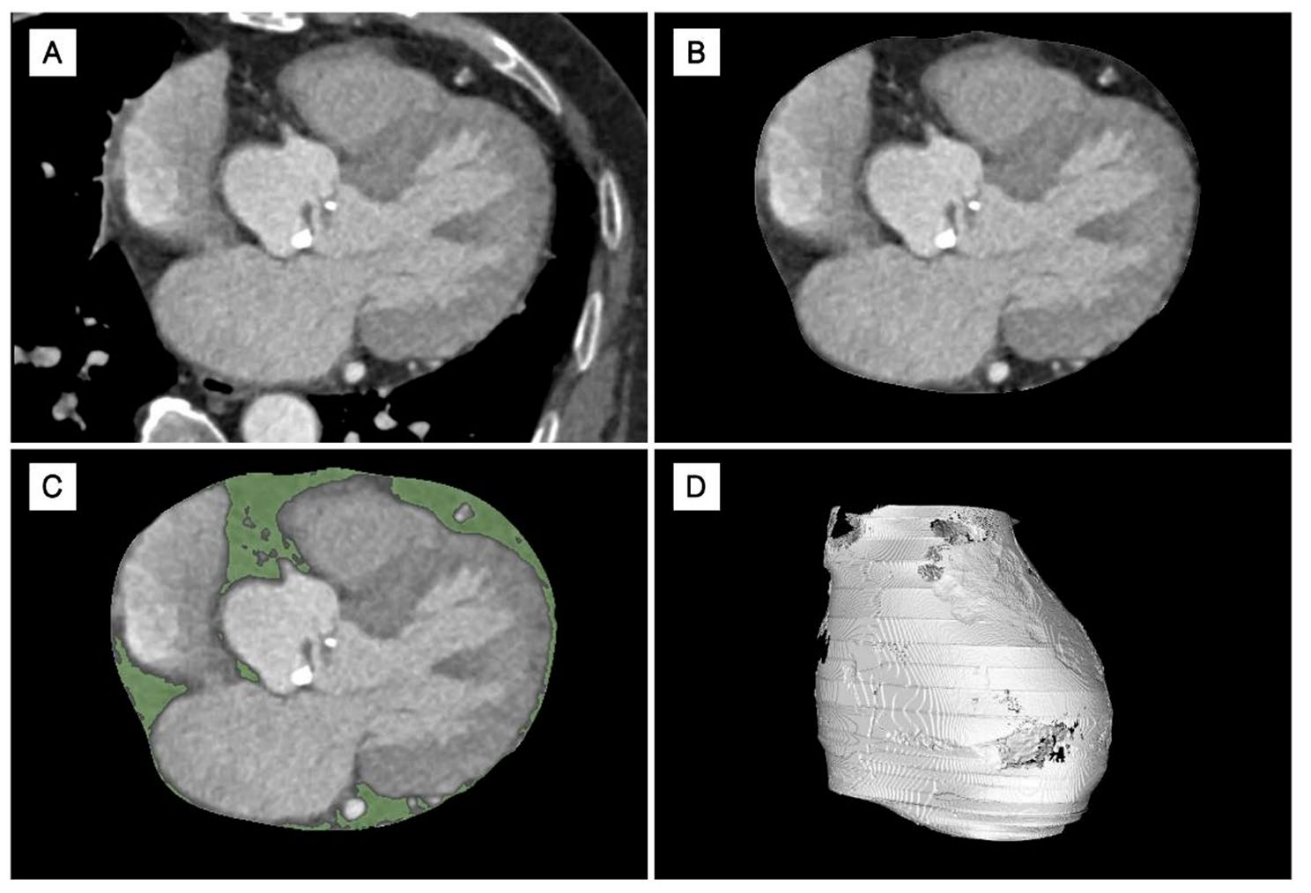
Measurement of EAT on CT imaging. Panel (A) manual contour of the pericardial border; Panel (B) automatic exclusion of structures outside the contour by the software; Panel (C) identification of the range of attenuation between − 10 and − 190 HU automatically by the software; Panel (D) The 3D reconstruction of the EAT layer and its volume was automatically obtained by multiplying the sum of all measured areas by the slice thickness

Pre- procedural MSCT was done for procedural planning while post-procedural MSCT was done to monitor structural valve deterioration. All MSCT scans were contrasted and performed with a second-generation dual-source CT system (SIEMENS SOMATOM Definition Flash; SIEMENS Healthcare, Erlangen, Germany). EAT volume and the average attenuation were semi-automatically quantified on MSCT using a commercially available software, Horos software ver. 2.0.2 (Horos Project, Annapolis, MD, USA). For each CT image, several contours were manually created to trace pericardial borders on axial images started at the level of the pulmonary trunk and ended at the level of the inferior diaphragmatic surface of the heart. The software then automatically generated missing borders that can be manually adjusted. The area outside the traced pericardium was excluded. The range of attenuation for EAT segmentation was then set between − 10 and − 190 HU [14], which allowed the exclusion of other tissues in the region of interest (Fig. 1). Total EAT volume was automatically obtained by multiplying the sum of all measured areas by the slice thickness and the overall average CT attenuation was calculated as the mean of all EAT voxels’ attenuation automatically by the software.

### Statistics

Continuous variables are presented as mean ± standard deviation or median (interquartile range, IQR). Categorical variables were presented as frequencies and percentages. Comparisons were performed in baseline and clinical characteristics, echocardiographic and computed tomographic measurements, as well as procedural and post-procedural details between the two groups divided by LVMi change. Differences in proportions were assessed using the Chi-squared test or Fisher’s exact test while differences in means between groups with continuous variables were assessed using an unpaired Student’s t test; or when the variables were not normally distributed, Mann–Whitney non-parametric test was used to test ranks. If statistically significant, a multivariate logistic regression with variables that were different between groups was then performed adjusted by age and sex. The sufficiency of data had been taken into consideration and Hosmer-Lemeshow test was used to assess the model’s goodness-of-fit. All computations relied on a commercially available software, SPSS version 21.0 software (IBM, Chicago, Illinois, USA), with statistical significance set at two-tailed 0.05.

## Results

A total of 152 patients were included. The median age of these patients was 74 years (IQR 69–78 years), and male (n = 86) accounted for 56.6%. LVMi decreased (163.7 [IQR:133.9-194.3] g/m^2^ vs. 119.2 [IQR:102.3-144.6] g/m^2^, P < 0.0001) significantly one year post-TAVR, but 28.9% of patients had inadequate LVMi regression (17.8% of patients had a decrease less than 15% while 11.2% of patients had an increase of LVMi).The median EAT volume at baseline was 108.0 cm^3^ (IQR 85.9-139.4 cm^3^) and 112 patients had an increase in EAT volume at one-year follow-up with a medianΔEAT volume (i.e. 1-year EAT volume – baseline EAT volume) of 16.7 cm^3^ (IQR − 0.7–24.9 cm^3^). Meanwhile, the average CT attenuation of EAT at baseline was − 63.07 ± 9.66 HU and 119 patients had an decrease in average CT attenuation of EAT at one-year follow-up with a meanΔEAT attenuation (i.e. average CT attenuation of EAT at 1-year follow-up – average CT attenuation of EAT at baseline) of -7.46 HU (95% CI: -8.97 to -5.96 HU).

Between groups of adequate (i.e. a decrease of LVMi ≥ 15%) and inadequate (i.e. a decrease of LVMi < 15%) LV reverse remodeling, baseline atrial fibrillation (10.2% vs. 22.7%, P = 0.042), mitral regurgitation ≥ mild (63.0% vs. 38.6%, P = 0.006), aortic regurgitation (AR) > mild (43.5% vs. 15.9%, P = 0.001) as well asΔEAT attenuation (-8.40 ± 10.08 HU vs.-5.16 ± 6.93 HU,P = 0.026) were statistically different (Table 1). In the multivariate regression adjusted by age and sex (Table 2), more than mild AR at baseline remained as the only statistically significant predictor of the decrease of LVMi < 15% at one year (OR 0.33, 95% CI: 0.13 to 0.84, P = 0.021). Hosmer-Lemeshow test showed a satisfying goodness-of-fit of this model (P = 0.378).

**Table 1:** Difference between groups in baseline characteristics, procedural details and echocardiographic data

	Adequate LVMi regression N = 108		Inadequate LVMi regression N = 44	P value
**Baseline characteristics**				
Age (year)		74 (68–78)	74 (70–80)	0.138
Male, n (%)		62 (57.4)	24 (54.5)	0.747
Height (cm)		1.60 (1.53–1.68)	1.60 (1.55–1.68)	0.658
Weight (kg)		57.6 ± 9.5	58.0 ± 8.5	0.816
BMI (kg/m²)		22.6 ± 3.4	22.4 ± 3.0	0.831
BSA (m²)		1.68 ± 0.15	1.69 ± 0.14	0.680
STS Score (%)		6.82 (4.19–9.25)	5.82 (4.04–8.51)	0.320
NYHA class III-IV, n (%)		97 (89.8)	39 (88.6)	0.939
Hypertension, n (%)		41 (38.0)	18 (40.9)	0.735
Diabetes, n (%)		18 (16.7)	11 (25.0)	0.236
COPD, n (%)		64 (59.3)	23 (52.3)	0.430
AF, n (%)		11 (10.2)	10 (22.7)	0.042
CAD, n (%)		36 (33.33)	17 (38.64)	0.534
Previous PCI, n (%)		9 (8.3)	4 (9.1)	0.866
Previous MI, n (%)		2 (1.9)	0 (0.0)	0.999
CVD, n (%)		21 (19.4)	13 (29.6)	0.175
eGFR (ml/min/1.73m^2^)		49.6 (38.0-65.2)	51.7 (40.9–61.5)	0.521
**Echocardiography**				
MS ≥ mild, n (%)		7 (6.5)	3 (6.8)	0.776
MR ≥ mild, n (%)		68 (63.0)	17 (38.6)	0.006
AR >mild, n (%)		48 (44.4)	7 (15.9)	0.001
LVEF (%)		58.0 (43.0–68.0)	62.5 (55.0-70.5)	0.064
Mean PG (baseline, mmHg)		63.0 (51.0–82.0)	57.0 (47.0-71.8)	0.132
Mean PG (1-year post-TAVR, mmHg)		11.0 (8.0–15.0)	11.0 (8.0-15.8)	0.847
**MSCT**				
Bicuspid valve, n (%)		56 (51.9)	29 (65.9)	0.113
Annulus perimeter (cm)		78.8 (72.6–84.0)	73.6 (70.6–84.3)	0.193
LVOT perimeter (cm)		81.2 (73.3–91.5)	77.2 (70.4–86.3)	0.105
STJ perimeter (cm)		95.8 (87.8-105.8)	93.80 (88.7-104.2)	0.563
SOV perimeter (cm)		109.6 (101.8-118.5)	110.4 (98.6-117.2)	0.517
EAT volume (Baseline, cm^3^)		107.3 (77.0-141.6)	109.4 (91.0-137.5)	0.536
EAT volume (1-year post-TAVR, cm^3^)		133.2 ± 54.5	127.3 ± 43.8	0.591
ΔEAT volume (cm^3^)		19.0 (1.0-34.2)	10.1 (-5.0-23.4)	0.092
EAT attenuation (Baseline, HU)		-62.36 ± 9.84	-64.96 ± 9.12	0.138
EAT attenuation (1-year post-TAVR, HU)		-70.76 ± 8.37	-70.12 ± 8.36	0.669
ΔEAT attenuation (HU)		-8.40 ± 10.08	-5.16 ± 6.93	0.026
**Procedural details**				
Pre-dilation, n (%)		98 (90.7)	39 (88.6)	0.923
Post-dilation, n (%)		50 (46.3)	22 (50.0)	0.678
s valve implantation, n (%)		11 (10.2)	4 (9.1)	0.925
**Post-procedural outcomes**				
PVL ≥ mild, n (%)		33 (30.6)	9 (20.5)	0.207
New LBBB, n (%)		32 (29.6)	16 (36.4)	0.418
PPI, n (%)		23 (21.3)	13 (29.6)	0.278

Values are mean ± SD, n (%), or median (interquartile range). BMI = body mass index; BSA = body surface area; STS = Society of Thoracic Surgeons; NYHA = New York Heart Association; COPD = chronic obstructive pulmonary disease; AF = atrial fibrillation; CAD = coronary artery disease; PCI = percutaneous coronary intervention; MI = myocardial infarction; CVD = cerebrovascular disease; eGFR = evaluated glomerular filtration rate; MS = mitral stenosis; MR = mitral regurgitation; AR = aortic regurgitation; LVEF = left ventricular ejection fraction; PG = pressure gradient of aortic valve; LVOT = left ventricular outflow tract; STJ = sinutubular junction; SOV perimeter = perimeter sinus of Valsalva; EAT = epicardial adipose tissue; ΔEAT volume = EAT volume (1-year follow-up)- EAT volume (Baseline); PVL = perivalvular leakage; LBBB = left bundle branch block; PPI = permanent pacemaker implantation

**Table 2:** Multivariate regression for inadequate LV reverse remodeling post-TAVR

	Univariate analysis			Multivariate analysis	
	OR (95% CI)		P value	OR (95% CI)	P value
Age (years)		1.05(0.99–1.11)	0.084	1.04(0.97–1.10)	0.269
Sex (male)		0.89(0.44–1.80)	0.747	0.94(0.43–2.06)	0.878
AF		2.60(1.01–6.65)	0.047	2.22(0.81–6.11)	0.123
MR (≥ mild)		0.37(0.18–0.76)	0.007	0.55(0.25–1.21)	0.138
AR (>mild)		0.24(0.10–0.58)	0.002	0.33(0.13–0.84)	0.021
ΔEAT attenuation (HU)		1.04(0.99–1.08)	0.058	1.02(0.97–1.06)	0.433

CI = confidence interval; OR = odds ratio; other abbreviations as in Table 1

## Discussion

The main findings of our study are that (1) LVMi underwent a more pronounced decrease at one year when AS is concomitant with more than mild AR at baseline in TAVR recipients. (2) EAT, being recognized to exert endocrine and paracrine effects [15], also had a dynamic change post-TAVR.

It is known that isolated AS exerts only pressure overload to the LV myocardium while AS concomitant with AR exerts both pressure and volume overload. This different pathologic adaptation might lead to different structural changes of LV following treatment. In a previous study, *E. Mara Vollema et al.* found the decrease in LVMi was more pronounced in patients with isolated AR during the first year after surgical AVR than isolated AS [16]. Thus, we assumed that the different pattern of LV remodeling caused by coexisting AR contributes to, at least partly, a different time course of LV reverse remodeling in TAVR recipients. Moreover, from an outcome perspective, *Johnny Chahine et al.* have reported a better overall survival in patients with AS accompanied by AR compared to patients with isolated AS [17]. It is likely that the different response of LV reverse remodeling from TAVR might be a possible contributor to this survival benefit brought by coexisting AR as a more pronounced decrease in LVMi has been associated with better prognosis [5, 18]. Although PVL can also exert volume overload to the LV myocardium, it differs from AR at baseline as it is a remaining or newly emerged hemodynamic change after TAVR in the absence of AS. Post-TAVR PVL in this cohort, however, did not result in different LVMi. There might be two possible explanations. It is noteworthy that we conducted statistical analyses of PVL that was mild and above (mild in 33 cases and mild-to-moderate in 9 cases) while only more than mild AR was included in statistical analyses (mild-to-moderate in 14 cases and moderate or above in 41cases). The reason that we include mild PVL in the PVL group is that it is now recognized that even mild PVL affects outcomes after TAVR [19]. Therefore, it is obvious that the regurgitant volume of PVL cases was much smaller than cases with AR at baseline. Besides, the duration of volume overload caused by PVL was much shorter than hemodynamic disorders caused by AR at baseline as well.

In addition, our analysis adds the little evidence existed for the dynamic change of EAT post-TAVR. We observed an overall increase in EAT volume and an overall decrease in the average CT attenuation of EAT during 1-year follow-up from CT imaging post-TAVR, although these changes failed to be correlated with clinical outcomes. It has been shown that there is no anatomical barrier between EAT and the adjacent heart structures [20], which allows crosstalk between the two. EAT can exhibit either protective or harmful property for the underlying myocardium in different circumstances [21], and therefore, it has been suggested to play a part in several heart diseases. In heart failure, EAT is involved in the development and progression of disease through several mechanisms including increased inflammation and fibrosis, autonomic dysregulation and mechanic effects [22]. A strong correlation has been demonstrated between an increased EAT volume and worsening left ventricular diastolic relaxation and filling [23, 24]. A larger EAT volume at baseline has been previously associated with a worse prognosis after TAVR (an increased all-cause 1-, 2-, and 3-year mortality and an increased risk to reach the early safety endpoint) [11], although the study did not monitor volumetric changes of EAT during follow-up. Besides volume, CT attenuation might also be an important parameter of EAT, given that CT attenuation of EAT is determined by the adipocyte size/adipose tissue lipid content and interstitial fibrosis [22, 25]. In coronary artery disease, fat attenuation index (FAI), which is defined as the average attenuation of adipose tissue within a volume of interest as measured from reconstructed CT, was first proposed by *Alexios S. Antonopoulos et al.* as a marker of perivascular adipose tissue (PVAT) inflammation [25]. In their research, the differentiation of preadipocytes and intracellular lipid accumulation were found to be restrained by the contiguous vascular inflammation, which could be visualized as an increase in the attenuation of corresponding PVAT on CT imaging [25]. Therefore, FAI could be used to detect vascular inflammation and discriminate stable and unstable coronary plaques. The perivascular FAI has been translated into clinical practice for its ability to predict and stratify cardiac risk and a high perivascular FAI value (cutoff ≥-70.1 HU) has been found as an indicator of increased cardiac mortality [26]. The decrease in the average CT attenuation observed in our research might also suggest a shift in the property of EAT. However, FAI is a relatively precise measurement to evaluate adipose tissues surrounding the vessel within millimeters but we only measured a rough average attenuation of the whole EAT surrounding the heart. The fact that this rough value decreased following TAVR merits further identification of regional property indicators of EAT besides its overall volume change. Neither the change in volume nor in CT attenuation of EAT was found to affect clinical outcomes in the current analysis. It is possible that at one year post-TAVR, the relief of pressure overload remains the main player that impacts LV reverse remodeling and the effect of EAT changes on LV myocardium might emerge afterwards, thus longer follow-up is warranted to understand the corresponding consequences.

Our results have limitations inherent to single-center retrospective studies, including a small sample size, selection and treatment bias, and the absence of blinded core-laboratory adjudicated echocardiographic and MSCT measurements. There could be a survival bias because the data in this study are collected from patients who completed one-year echocardiographic and MSCT follow-ups.

## Conclusion

Patients underwent TAVR with pre-existing > mild AR appeared to experience a more pronounced LV reverse remodeling at mid-term.

## Data Availability

The datasets used and/or analyzed during the current study are available from the corresponding author on reasonable request.

## Abbreviations

AF: atrial fibrillation
AR: aortic regurgitation
AS: aortic stenosis
BMI: body mass index
BSA: body surface area
CAD: coronary artery disease
CI: confidence interval
COPD: chronic obstructive pulmonary diseaseCVD cerebrovascular disease
CVW: vena contracta width
EAT: epicardial adipose tissue
eGFR: evaluated glomerular filtration rate
FAI: fat attenuation index
IQR: interquartile range
IVSd: intraventricular septum in diastole
LBBB: left bundle branch block
LV: left ventricular
LVIDd: left ventricular inner diameter in diastole
LVMi: left ventricular mass index
LVOT: left ventricular outflow tract
LVPWd: left ventricular posterior wall in diastole
MI: myocardial infarction
MR: mitral regurgitation
MS: mitral stenosis
MSCT: multi-slice computed tomography
NYHA: New York Heart Association
OR: odds ratio
PCI: percutaneous coronary intervention
PG: pressure gradient of aortic valve
PPI: permanent pacemaker implantation
PVL: paravalvular leakage
PVAT: perivascular adipose tissue
SOV: sinus of Valsalva
STJ: sinutubular junction
STS: Society of Thoracic Surgeons
TAVR: transcatheter aortic valve replacement
TTE: Transthoracic echocardiography
